# A 61-Year-Old Man with Dyspepsia and Weight Loss

**DOI:** 10.1371/journal.pmed.0020154

**Published:** 2005-06-28

**Authors:** Catherine Burton

## Abstract

In this case-based Learning Forum, Burton takes clinicians through the key steps in diagnosing and managing this patient.

## DESCRIPTION of CASE

A 61-year-old male carpenter presented with ten months' history of dyspepsia. More recently he had developed epigastric pain and had lost four kilograms in weight because of anorexia. He was otherwise asymptomatic and in particular there was no history of nausea, vomiting, abdominal distension, melaena, haematemesis, diarrhoea, constipation, night sweats, or fevers. There was no past medical history of note, and he had a performance status of zero (see Table 1). His only medication was lansoprazole. He drank no alcohol and smoked 40 cigarettes/day. Examination was unremarkable. All blood tests, including full blood count, biochemistry, and lactate dehydrogenase, were normal.

**Table 1:**
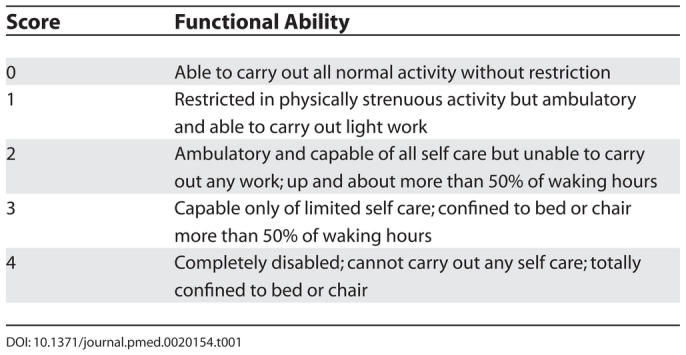
World Health Organization Performance Status

### What Was the Differential Diagnosis at This Point?

The differential diagnosis in a patient with dyspepsia, epigastric pain, and weight loss is gastro-oesophageal reflux, gastritis, gallstones, peptic ulcer, oesophageal/gastric carcinoma, lymphoma, and inflammatory bowel disease.

The patient underwent an endoscopy which showed a 4–5-cm ulcer with rolled edges on the greater curve of the antrum of the stomach. Appearances were suggestive of a malignant lesion.

### What Was the Most Likely Diagnosis?

The likely diagnosis was therefore either gastric carcinoma or lymphoma—most likely non-Hodgkin lymphoma (NHL)—specifically, either gastric mucosa-associated lymphoid tissue (MALT) lymphoma or diffuse large B cell lymphoma (DLBCL).

**Figure 1:**
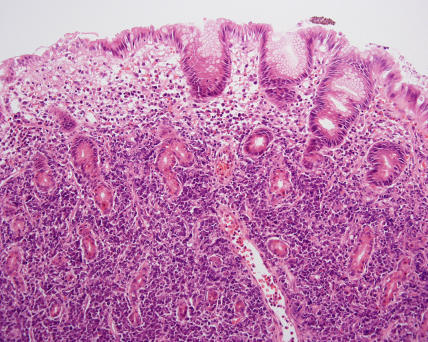
Low-Power Haematoxylin and Eosin Staining Showing DLBCL DLBCL is composed of large transformed lymphoid cells, with oval to round nuclei with fine chromatin and membrane-bound nucleoli. Surface and/or cytoplasmic immunoglobulin (IgM > IgG > IgA) is demonstrated in 50%–75% of cases. BCL-2 is positive in approximately 30%–50% of cases and is associated with a poorer prognosis.

**Figure 2:**
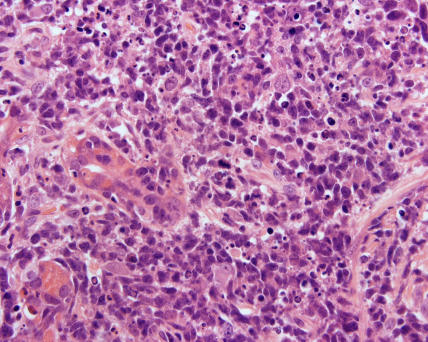
High-Power Haematoxylin and Eosin Staining Showing DLBCL

**Figure 3:**
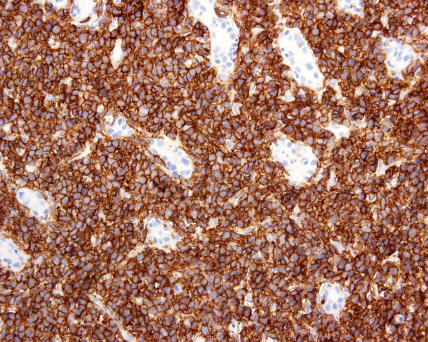
Strong CD20 Positivity DLBCL usually expresses various pan-B markers, e.g. CD20 and CD79a.

**Figure 4:**
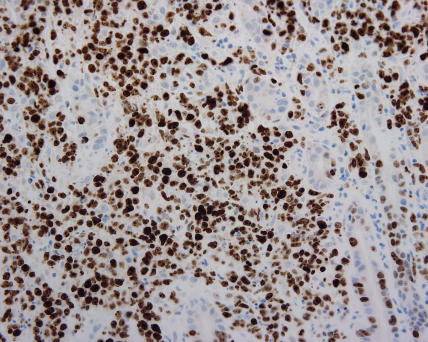
High MIB-1 Expression MIB-1 staining demonstrates proliferative activity of DLBCL. Proportion of cells stained is usually greater than 40%.

Histology revealed a diagnosis of DLBCL (see Figures 1 and 2). The biopsy was positive for surface markers CD20 and CD79a and surface immunoglobulin (IgM). These markers are found on B cells, and positivity confirmed the B cell origin of the lymphoma (see Figures 3 and 4). The biopsy was negative for BCL-2 protein expression. Positive BCL-2 expression is associated with an adverse prognosis. Staining for Helicobacter pylori antibody was negative.

### Which Further Investigations Were Indicated?

**Figure 5:**
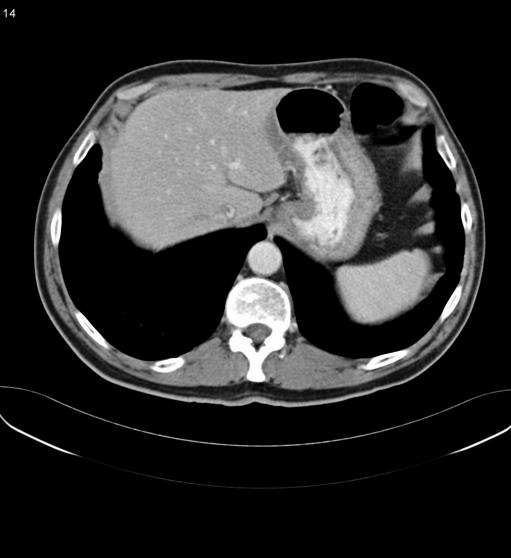
CT at Diagnosis Showing Thickening of Gastro-Oesophageal Junction and Stomach Wall

In view of the diagnosis of lymphoma, staging investigations were performed. Computed tomography (CT) imaging showed thickening of the gastro-oesophageal junction (see Figure 5). Multiple subcentimetre lymph nodes within the abdomen were also noted, but these were not enlarged by CT criteria. CT chest was normal. A bone marrow biopsy was normal.

The patient was therefore diagnosed with stage IE (see Table 2) DLBCL of the stomach. His international prognostic index (IPI) score was one (see Table 3).

**Table 2:**
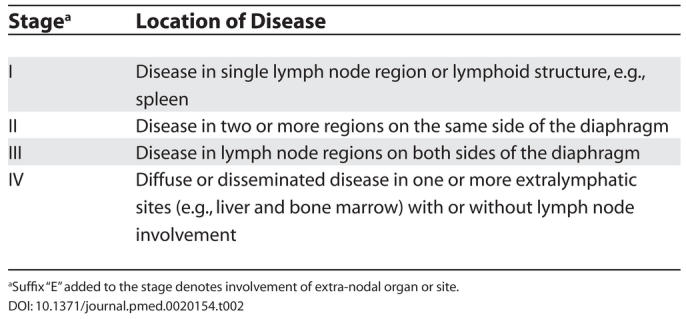
Ann Arbor Staging System

^a^Suffix “E” added to the stage denotes involvement of extra-nodal organ or site.

**Table 3:**
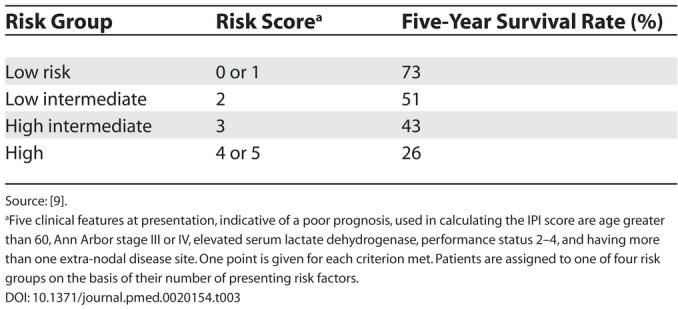
International Prognostic Index

Source: [9].

^a^Five clinical features at presentation, indicative of a poor prognosis, used in calculating the IPI score are age greater than 60, Ann Arbor stage III or IV, elevated serum lactate dehydrogenase, performance status 2–4, and having more than one extra-nodal disease site. One point is given for each criterion met. Patients are assigned to one of four risk groups on the basis of their number of presenting risk factors.

### What Was the Appropriate Treatment?

**Figure 6:**
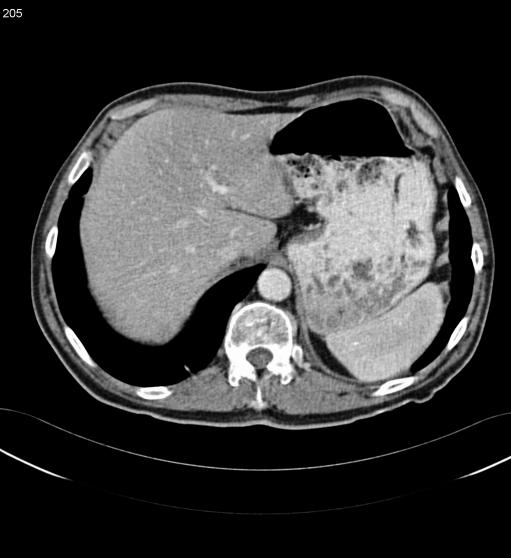
Post-Treatment CT Showing Almost Complete Resolution of the Abnormal Thickening of Gastro-Oesophageal Junction and Stomach Wall

The patient was treated with R-CHOP chemotherapy (rituximab, cyclophosphamide, doxorubicin, vincristine, and prednisolone). He received four initial courses administered once every three weeks. His chemotherapy course was complicated by a hospital admission with Klebsiella pneumonia that responded well to antibiotics. After four courses of treatment he underwent a restaging CT and endoscopy. The CT scan showed improvement in the thickening of the gastro-oesophageal junction and no significant lymphadenopathy (see Figure 6). The repeat endoscopy and biopsy were normal.

The patient went on to have two further courses of consolidation R-CHOP chemotherapy. Repeat CT imaging after six courses of chemotherapy showed no further change in the thickening of the gastro-oesophageal junction. In view of the fact that there had been no further response between the fourth and sixth courses of chemotherapy and a biopsy had been negative, no further consolidation chemotherapy was given. The final CT also showed collapse of the left lower lobe of the patient's lung. In view of these findings and the patient's history of being a heavy smoker, he underwent a fibre optic bronchoscopy. This revealed a mucus plug at the orifice of the left lower lobe, probably secondary to his previous pneumonia. The mucosa was normal. A repeat chest X ray showed re-inflation of the left lower lobe of his lung.

At follow-up this patient remains well and asymptomatic and is gaining weight. His last blood tests were normal. He remains under standard review every three months.

## DISCUSSION

Symptoms of dyspepsia and epigastric pain are common, especially in the Western world. There is a one in ten lifetime risk for people in the Western world of developing a peptic ulcer [1]. If symptoms persist or clinical history is suggestive of significant pathology, an endoscopy with biopsies is mandatory. Although not at the top of the list of differential diagnoses, this case highlights the fact that lymphoma needs to be considered as a possible diagnosis.

### Non-Hodgkin Lymphoma

NHL is the 11th commonest malignancy worldwide. It is increasing in incidence, with more than 300,000 cases worldwide and 9,000 cases in the United Kingdom diagnosed each year [2]. DLBCL is the most frequently occurring NHL, constituting about one-third of all adult NHL [3]. There is a slight male predominance, and median age of presentation is in the seventh decade [3,4], as in this patient, although the age range is broad.

Patients can present with nodal or extra-nodal disease. Up to 40% of cases are initially confined to extra-nodal sites [5]. The most common extra-nodal site is the gastrointestinal tract [5], especially the stomach, although any extra-nodal location can be a primary site. Lymphomas of the stomach can either be indolent MALT lymphoma or DLBCL, which may or may not be on the background of a MALT lymphoma.

For MALT lymphoma the median age of presentation is in the seventh decade—median age is 61 years—with a slight female predominance [4]. There is a close association between gastric MALT lymphoma and Helicobacter pylori infection; over 90% of cases are H. pylori positive [6]. For MALT lymphoma, if the lesion is limited to the gastric mucosa, eradication of the bacteria with triple antibiotic therapy is often sufficient to cause regression of the lymphoma [7,8], whereas DLBCL also requires treatment with chemotherapy.

Use of the IPI score [9], defined by clinical prognostic factors, is currently the standard approach to stratify patient risk in DLBCL (see Table 3). Difference in outcome, though, between groups of patients with similar IPI scores indicates that DLBCL encompasses a clinically and biologically heterogeneous group of tumours, which vary in response to chemotherapy. Combining the IPI risk score with cellular and molecular markers may improve patient risk stratification at presentation. For example, germinal cell phenotype, defined by BCL-6 and CD10 positivity, has been associated with a better outcome [10,11], and BCL-2 protein expression has been shown to be predictive of a worse outcome [11–15]. Both have been demonstrated immunohistochemically and confirmed by gene microarray analysis [16–18]. Combining cellular prognostic factors with the IPI is likely to become increasingly important in predicting outcome and tailoring therapy for DLBCL [19].

### Treatment of DLBCL

DLBCL is an aggressive lymphoma but potentially curable. Treatment for DLBCL is combination chemotherapy plus the monoclonal antibody rituximab [20]. CHOP chemotherapy (cyclophosphamide, doxorubicin, vincristine, and prednisolone) is given every three weeks. Rituximab is a chimeric monoclonal antibody containing human IgG lambda and kappa constant regions with murine variable regions, which has a high affinity for the CD20 antigen. A dose of 375 mg/m^2^ given every three weeks with CHOP chemotherapy is now considered standard therapy for DLBCL. The mechanisms of action of anti-CD20 antibody are multiple and include induction of antibody-dependent cell-mediated cytotoxicity, induction of complement-mediated lysis, induction of apoptosis, and sensitisation of resistant lymphoma cells to chemotherapeutic agents.

Rituximab and CHOP chemotherapy have non-overlapping toxic effects, with emerging evidence of synergy [20,21]. The French GELA group (Groupe d'Etude des Lymphomes de l'Adulte) showed that addition of rituximab to CHOP (R-CHOP) had significant activity in elderly patients newly diagnosed with DLBCL [20]. CHOP plus rituximab was associated with significantly better overall response rate (94%), complete response rate (76%), event-free survival, and overall survival than CHOP alone. This survival advantage in elderly patients (>60 years) with DLBCL following R-CHOP represents the first major therapeutic advance for this group of patients since the introduction of CHOP nearly 30 years ago. Recent studies show similar benefits in younger patients [21,22], so rituximab in combination with CHOP should be used for all newly diagnosed patients with DLBCL.

### Toxicity of Chemotherapy

General toxicity associated with chemotherapy includes mucositis, gastrointestinal disturbance, myelosuppression (increasing the risk of infections and bleeding), alopecia, parathesiae, and infertility. Specific side effects of chemotherapeutic agents used for treating NHL are shown in Table 4.

**Table 4:**
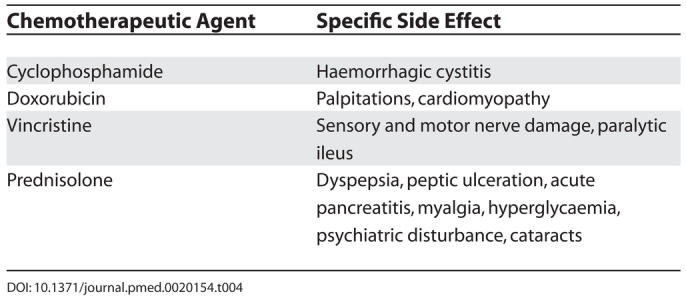
Specific Side Effects of Chemotherapeutic Agents Used for Treating NHL

Side effects of rituximab include fevers, headache, nausea, lethargy, myalgia, arthralgia, skin erythema, pruritus, and hypotension. Most of these symptoms disappear upon temporary slowing or discontinuation of the treatment or after the administration of paracetamol and/or anti-allergic medication. Fatalities following severe cytokine release syndrome—which is characterised by severe shortness of breath and associated with features of tumour lysis syndrome—have occurred 1–2 hours after infusion of rituximab([23], Section 8.2.3; [24]). Patients with a high tumour burden as well as those with pulmonary insufficiency or infiltration are at increased risk and should be monitored very closely, and a slower rate of infusion should be considered as necessary.

Related to this case in particular, patients with gastric ulcers are at risk of perforation on initiation of treatment. Such patients should receive proton-pump inhibitor prophylaxis during chemotherapy. There is a need for close observation when chemotherapy is started, but the presence of a gastric ulcer is not an indication for dose reduction.

### Monitoring Response to Chemotherapy

Response to chemotherapy needs to be monitored on a regular basis. Patients should be restaged after four courses of R-CHOP chemotherapy with CT scanning. If the bone marrow was involved at diagnosis, then a repeat biopsy is required. In cases such as our patient where disease was apparent elsewhere, a repeat biopsy of that lesion should be performed.

In the UK, standard practice for those in complete remission with no evidence of disease is to receive a further two courses of R-CHOP consolidation. Those in partial remission (≥50% improvement) after four courses are restaged after six courses, and if they are improving they go on to receive two further courses of R-CHOP. If there is no further improvement, consolidation radiotherapy is considered. Those in partial remission after four courses with no further improvement after six courses of R-CHOP receive consolidation radiotherapy or are considered for second-line treatment with an alternative regimen containing different chemotherapeutic agents.

Practice elsewhere in Europe varies, with up to eight courses of R-CHOP being given regardless of remission status. Although not universally available, FDG-PET (2-fluoro-2-deoxy-d-glucose positron emission tomography) is a functional imaging technique that is currently the most accurate modality to identify residual disease and differentiate it from scar tissue in patients with residual radiological abnormalities [25–27]. In general, re-biopsy of suspicious lesions should be considered.

**Learning Points**

- In all patients a careful history of the nature of the dyspepsia and the associated symptoms should be taken. All patients over 45 years with dyspepsia should have an endoscopy performed. For younger patients, those with persistent symptoms or clinical history suggestive of significant pathology such as weight loss should also undergo endoscopy.
- Not all gastric lesions require complete surgical resection. It is imperative that an endoscopy with biopsies is performed to allow histological diagnosis.
- Patients with DLBCL require staging to ascertain the extent of disease and for comparison with post-therapy imaging. Determination of the IPI score is important to assign risk group and to predict prognosis.
- Optimal treatment for DLBCL is CHOP chemotherapy in combination with rituximab. Patients should receive 6–8 courses.

## Abbreviations

CT: computed tomography
DLBCL: diffuse large B cell lymphoma
IPI: international prognostic index
MALT: mucosa-associated lymphoid tissue
NHL: non-Hodgkin lymphoma
